# An evidence‐based structured one‐year programme to sustain physical activity in patients with heart failure in primary care: A non‐randomized longitudinal feasibility study

**DOI:** 10.1002/nop2.510

**Published:** 2020-05-15

**Authors:** Lena Nordgren, Anne Söderlund

**Affiliations:** ^1^ Centre for Clinical Research Sörmland Uppsala University Mälarsjukhuset Eskilstuna SE Sweden; ^2^ Department of Public Health and Caring Sciences Uppsala University Uppsala Sweden; ^3^ School of Health, Care and Social Welfare Mälardalen University Västerås Sweden

**Keywords:** feasibility study, heart failure, nursing, patients, physical activity, self‐efficacy

## Abstract

**Aim:**

The primary objective of this non‐randomized feasibility study was to test a 1‐year model programme for sustaining/increasing patients’ motivation to perform daily physical activity.

**Design:**

Non‐randomized longitudinal feasibility study with a one‐group repeated measures design.

**Methods:**

The study took place at a primary care centre in mid‐Sweden in 2017–2018. The model programme included individual and group‐based support, individualized physical activity prescriptions, a wrist‐worn activity tracker and an activity diary. The main outcomes were the participants’ perceptions of programme feasibility and scores on the Exercise Self‐Efficacy Scale.

**Results:**

Seven patients were recruited. Six patients completed the programme that was perceived to imply learning, motivation and support. Compared with baseline, the median score of the Exercise Self‐Efficacy Scale improved 3 months after participants completed the programme.

## 1 INTRODUCTION

Even though inactivity is associated with a statistically significant increase in all‐cause and cardiac death (Doukky et al., 2016), cardiac rehabilitation programmes continue to fail motivating patients with heart failure to sustain recommended levels of daily physical activity. Advantages of cardiac rehabilitation include improved muscle strength and condition, increased quality of life (Pihl, Cider, Strömberg, Fridlund, & Mårtensson, 2011) and reduced need for hospitalizations (Long et al., 2019; Pandey et al., 2015; Piepoli et al., 2011; Rajati et al., 2014). Improved physical capacity can have positive effects on heart failure symptoms (Anderson & Taylor, 2014; Lewinter et al., 2015) and mortality (Taylor et al., 2014); the positive effects of improved physical capacity can also be seen in older people (Schopfer & Forman, 2016). Physical activity also has positive effects on cognitive functions in older people and reduces the risk of falling (Schopfer & Forman, 2016). Nevertheless, many patients with heart failure do not follow recommendations for physical activity (Klompstra, 2016; Schopfer & Forman, 2016; van der Wal, van Veldhuisen, Veeger, Rutten, & Jaarsma, 2010; Yates, Pozehl, Kupzyk, Epstein, & Deka, 2017), and despite scientific evidence, physical activity is still underused as a form of treatment for people with heart failure.

### 1.1 Background

The reasoning behind the current study was based on several assumptions. People with heart failure can perceive low self‐efficacy in physical activity, which is associated with low actual levels of physical activity (Adsett, Morris, Kuys, Paratz, & Mudge, 2019). The condition in itself can also be perceived as an obstacle for physical activity (Klompstra, 2016; National Board of Health & Welfare, 2018); however, if people are motivated to perform physical activity, their self‐efficacy in physical activity will improve (Klompstra, 2016). Moreover, accurately accomplishing self‐management activities implicates high self‐efficacy (Boyne et al., 2014). Additionally, previous studies have found that exercise self‐efficacy is a strong predictor of physical activity in people with heart failure (Duncan, Pozehl, Hertzog, & Norman, 2014; Ha, Hare, Cameron, & Toukhsati, 2018; Pozehl, Duncan, Hertzog, & Norman, 2010; Yeh, Chan, Wayne, & Conboy, 2016). However, most clinical trials about physical activity for people with heart failure usually have been on short‐term interventions, that is interventions that last for 12–24 weeks (e.g. Abdelbasset & Alqahtani, 2019; Adsett et al., 2019; Duncan et al., 2014; Hagglund, Hagerman, Dencker, & Stromberg, 2017; Maru, Mudge, Suna, Scuffham, & Investigators, 2019; Pozehl et al., 2010; Teng, Yeh, & Wang, 2018; Xueyu, Hao, Shunlin, Rongbin, & Yuan, 2017; Yeh et al., 2016). Even though there are some long‐term intervention studies, that is 6–24 months of physical activity (e.g. Du et al., 2018; Evangelista, Cacciata, Stromberg, & Dracup, 2017; Howden et al., 2018), the knowledge about self‐efficacy in long‐term exercise behaviour is sparse. Hence, the novel aspect of the current study was a long‐term model for an intervention programme where the starting point of the content was based on current research findings.

The point of departure for the present study, then, was an idea that participation in a long‐term intervention programme that involves structured, tangible and clear advice and support can motivate people with heart failure to perform regular physical activity (Dontje et al., 2014; Jaarsma et al., 2015), eventually change their physical activity behaviour and maintain it. Current research findings imply that there might be enough evidence for setting up and implementing evidence‐based programmes for cardiac rehabilitation for patients with heart failure. However, with regard to optimize outcomes and to refine the content and structure of such programmes, randomized controlled trials (RCTs) should still precede the implementation, and before an RCT is undertaken, the feasibility of the model programme should be tested and evaluated.

Thus, the present study was a feasibility study conducted in advance of a future RCT. The objective was to study the feasibility of an evidence‐based structured 1‐year programme for physical activity for people with heart failure. The specific aims were to study the effects of the programme on physical activity levels, self‐efficacy in physical activity, quality of life, symptoms of heart failure, health‐related quality of life and low mood. The research question was whether an evidence‐based structured 1‐year programme for physical activity for people with heart failure could be feasible and possibly imply for improvement in the selected outcomes.

### 1.2 Design

This was a non‐randomized longitudinal feasibility study with a one‐group repeated measures design conducted at a primary care centre in a mid‐sized town in Sweden. The study conforms to the CONSORT 2010 statement: extension to randomized pilot and feasibility trials (Eldridge etal.,^2016^).The study was registered in the Open Science Framework (Nordgren, 2020).

## 2 METHODS

### 2.1 Development of the model programme

A broad scoping review was conducted prior to the feasibility study to identify available evidence that could serve as a basis for the model programme. These research findings were then used in the development of the programme.

Low levels of activity and reduced self‐management ability are often associated with insufficient knowledge (Lockhart, Foreman, Mase, & Heisler, 2014). In turn, insufficient knowledge is associated with recurrent hospitalizations and increased mortality (Lockhart et al., 2014). Therefore, a programme that aims to increase or sustain physical activity should contain continuous and repeated patient education and information (Boyne et al., 2014). It is important to discuss and provide information about lifestyle matters, since people with heart failure can have insufficient knowledge or delusive illness beliefs (Lockhart et al., 2014; MacInnes, 2014). It is equally important that care for older people with heart failure is performed by an interprofessional team that considers matters such as polypharmacy, nutrition and sleeping habits (Schopfer & Forman, 2016). Reoccurring medication reconciliations need too to be conducted (Schopfer & Forman, 2016). Hence, a structured programme that aims to increase or sustain participants’ level of physical activity should include all these prerequisites for affecting physical activity level in this specific target group. Thus, in our model programme, different members of an interprofessional team or other experts were invited to the group sessions to cover different themes (for instance, diet, medication, depression and self‐management). In addition, a free education written material (Active with heart failure; author's translation) from a non‐governmental organization, The Swedish Heart and Lung Association (2018), was used during some of the group sessions.

To facilitate people with heart failure to be more physically active, more than mere information about the value of physical activity is needed (Klompstra, 2016). Thus, the present programme included 10 group sessions in a separate room at a primary care centre. All group sessions were conducted in the afternoon and lasted between 1–2 hr. The presentation and discussion topics for the 10 group sessions were as follows: physical activity, diets, alcohol, tobacco use, depression/low mood, sleep disturbances, rest, behavioural changes, pharmacological treatment, the local county's heart failure process and basic knowledge about the condition and its effects on everyday life. During the group sessions, the participants were asked to reflect on what had happened since the last session. The participants had the opportunity to ask questions and to share their own experiences. Before the sessions were completed, there was a short summary and preparation for the next couple of weeks. To avoid misunderstandings and to take part in the participants’ experiences of the programme, the first author (LN) attended some of the group sessions.

Moreover, older people can perceive that they are lonely, socially isolated and perceive lacking control (Lockhart et al., 2014), so group‐based activities can offer an opportunity for social interaction with other people in a similar situation (Schopfer & Forman, 2016), which in turn can bring hope about their ability to manage their illness (Lockhart et al., 2014). In this programme, the participants met other patients in group sessions on a regular basis for a period of one year. Subsequently, the participants were able to encourage and support each other.

The scoping review also identified that regular assessments and self‐monitoring are important, for example, using an accelerometer (Deka, Pozehl, Williams, & Yates, 2016). If people can by monitoring gradually increase the intensity, duration and frequency of their activities, they will be more prepared to participate in future exercise or activity (Rajati et al., 2014). In the present study, the participants could choose between a simple pedometer and a wrist‐worn activity tracker (Deka, Pozehl, Norman, & Khazanchi, 2018); all chose the activity tracker to be worn during the one‐year program.

Activity diaries can facilitate behavioural changes (Deka et al., 2016). An activity diary can contain daily notes about the frequency, duration and intensity of activities (Deka et al., 2016). The existing resources in the primary care centre were limited, so a simple study‐specific exercise diary was developed and printed out on regular paper. The participants were encouraged to note what activities they had performed during the day and how many steps they had taken according to an activity tracker. There was also space for personal notes.

A central element that can contribute to successful rehabilitation is a person's belief in his or her ability to succeed, that is the person's self‐efficacy beliefs. The concept of self‐efficacy has been applied to studies on the rehabilitation of conditions such as musculoskeletal pain, whiplash‐associated disorders or obstructive sleep apnoea (Bring, Asenlof, & Soderlund, 2016; Denison, Asenlof, & Lindberg, 2004; Igelstrom, Emtner, Lindberg, & Asenlof, 2013; Soderlund & Asenlof, 2010); however, the concept is also increasingly used in relation to cardiovascular diseases (Tierney et al., 2012). Self‐efficacy is associated with mutual goal setting. In rehabilitation, this means that the patient and a healthcare professional formulate a goal together, for example for an activity or exercise, and the patient receives response and feedback for achieved or non‐achieved goals on a regular basis (Tierney et al., 2012). There are four main sources to increased self‐efficacy (Rajati et al., 2014): (a) the person has previous experiences of being successful; (b) the person has perceived that someone in a similar situation has succeeded; (c) the person receives direct encouragement from another person who is perceived as trustworthy; and (d) the person has positive experiences of physiological responses when conducting the task. To set realistic goals, the patient should have experiences of being previously able to succeed with the activity. The goal should be difficult enough that it is challenging, but it should also be possible to achieve. If patients manage to achieve their goals, their beliefs in their ability to succeed also improve, thus helping them achieve further goals. Thus, self‐efficacy‐increasing contents were included in the programme.

Previous studies have shown that a programme that aims to increase or sustain the participants’ level of physical activity should be individualized. That is, recommendations for activities and the frequency, intensity and duration of activities should be based on participants’ previous and existing activity habits (Deka et al., 2016; Klompstra, 2016; Klompstra, Jaarsma, & Stromberg, 2015) and their thoughts about the choice of an activity and their individual activity goals must be considered. To be perceived as pleasurable and satisfying, the programme should include alternatives to traditional exercises (Klompstra et al., 2015).

It is equally important that healthcare professionals and patients try to identify possible obstacles and available resources for physical activity (Davies et al., 2010; Klompstra, 2016; Klompstra et al., 2015). For people with heart failure, symptoms and fatigue can be perceived as obstacles (Klompstra, 2016). There can also be external obstacles, such as the absence of a nearby fitness centre, expenses, no interest or engagement from partners or friends, or poor weather conditions. However, internal factors such as a lack of time, a lack of knowledge, poor self‐discipline or a lack of motivation can also be perceived as obstacles (Klompstra, 2016). In the present study, the programme contained individual appointments in the first week, after 3 months, 6 months and 1 year with a Registered Nurse, a physiotherapist and a general practitioner from the interprofessional team who could provide support and encouragement. The nurse monitored the patients in accordance with ordinary standards for nurse‐led heart failure clinics in primary care (Liljeroos & Stromberg, 2019). The physiotherapist, who also was specialized in heart failure, regularly planned, followed and evaluated the participants’ progress with a 6‐min walking test and through dialogue with the participants about their subjective assessments (following Lockhart et al., 2014). They set goals and made plans for how to achieve those goals. The activity plan included the frequency, duration and intensity of activities. Later, the plan was revised if necessary. In accordance with the primary care centre's standard procedures, the participants had the opportunity to contact different team members between sessions if needed.

### 2.2 Sample

Patient enrolment was accomplished in August 2017. The model programme lasted for one year (Table 1). An interprofessional team specializing in heart failure care identified seven patients who were eligible for the study. The number of participants was based on the team's perceptions about what was reasonable for them to handle considering their overall work situation. A physician made a physical assessment about the study inclusion and exclusion criteria. The inclusion criteria were mild to moderate heart failure with reduced ejection fraction (EF < 50%). The exclusion criteria were the presence of symptoms of cognitive impairment or another severe disease with short expected survival. The eligible patients received oral and written information about the study and one of the nurses asked whether they wanted to participate; all patients agreed. They were asked to self‐monitor their physical activities daily in a diary. Data from the activity tracker and the diaries were not included in the data analyses.

**Table 1:** Summary table for programme components

Programme week	Individual appointment with nurse (RN), physiotherapist (PT), general practitioner (GP)	Group session leader: nurse (RN), physiotherapist (PT), general practitioner (GP), researcher (R)	Theme for group session (*N* = 10) 1–2 hr per session	Group sessions; detailed content	Individual activity
1	PT: Introduction to the programme. Agreement of individual activity plans including choosing activities and activity goal setting (frequency, duration, and intensity of activities). 6‐min walking test				Questionnaire^a^. Start self‐monitoring by activity tracker and diary
2		PT	Physical activity	Information and discussions about physical activity and heart failure	Self‐monitoring by activity tracker and diary
3					
4		RN	Follow‐up	Discussions and reflections over self‐monitored activities and related thoughts	–
5–6					–
7*		R	Dietician: Dietary^b^ RN: Alcohol, tobacco^b^	Discussions about food, dietary, alcohol, tobacco. Homework: What brings pleasure or joy in your life?	–
8–10					–
11		R	Psychologist: Depression/low mood, sleep disturbances, rest, behavioural changes	Lecture by psychologist and discussions. Discussions and reflections over matters that brings joy or pleasure	–
12	PT: Revision of individual activity plans and goals. 6‐min walking test. RN: Heart failure monitoring in accordance with ordinary standards				Questionnaire^a^. Self‐monitoring by activity tracker and diary
13–14					–
15		GP^b^	Pharmacist: Pharmacological treatment	Information, discussions and questions about medical treatment	–
16–22					–
23		RN	Regional process leader: The local county's heart failure process^c^	Information and discussions about the region‐wide heart failure process	–
24	PT: Revision of the individual activity plan and goals. 6‐min walking test. RN: Heart failure monitoring in accordance with ordinary standards				–
25–28					–
29		R	Active with heart failure 1: Getting to know the condition^d^	Basic knowledge that is needed for patients to understand and accept the illness. Discussions and reflections about self‐monitoring activities and related thoughts	–
30–36					–
37		R	Active with heart failure 2: Living with heart failure^d^	Basic knowledge about medical treatment and self‐monitoring activities. Practical tips about travelling. Discussions about being concerned and having experiences of low mood. Discussions and reflections about the self‐monitored activities and related thoughts	–
38–44					–
45		RN^b^	Active with heart failure 3: Daily choices^d^	Lifestyle matters, food and dietary, exercise and physical activity. Discussions and reflections about the self‐monitored activities and related thoughts	–
46–49					–
50		PT	Active with heart failure 4: Active with heart failure^d^	Repeat of course content, talking about the future. Discussions and reflections about the self‐monitored activities and related thoughts	–
51	PT: Revision of the individual activity plan and goals. Plan for future activity. 6‐min walking test. RN: Heart failure monitoring in accordance with ordinary standards. GP: Physical examination/health status				Questionnaire^a^
52		R	Research interview	Follow‐up; focus group interview	
66		R	Research interview	Follow‐up; individual interviews	Questionnaire^a^

^a^ Exercise self‐efficacy scale, level of physical activity, quality of life, health‐related quality of life, fatigue, dyspnoea, low mood.

^b^ Invited guest lecturers cancelled; researcher/RN replaced*

^c^ For more information, see Liljeroos & Strömberg, 2018.

^d^ Information in Swedish athttps://xn‐‐aktivmedhjrtsvikt‐zqb.se/skola/skola‐1/

### 2.3 Data collection

Sociodemographic data for date of birth, gender, annual income, level of education and occupation were provided by the participants. To evaluate the effects of the programme, the participants completed a self‐assessment questionnaire before the intervention (baseline), at 3, 6 and 12 months within the programme and 3 months after completing the programme. The questionnaire contained questions about self‐efficacy in physical activity (Exercise Self‐Efficacy Scale; 10 items, 4‐point Likert scale; Ahlström, Hellström, Emtner, & Anens, 2015; Kroll, Kehn, Ho, & Groah, 2007), level of physical activity during the last week (7‐choice question; modified from the Swedish short version of International Physical Activity Questionnaire; Craig et al., 2003; Ekelund et al., 2006), quality of life (10‐point visual analogue scale), fatigue and dyspnoea (4‐point Likert scale; modified from the Swedish Heart Failure Registry), health‐related quality of life (5 items, 3‐point Likert scale, modified from the Swedish Heart Failure Registry) and low mood (two items, 4‐point Likert scale; modified from the Swedish version of the Patient Health Questionnaire‐9). Physical capacity was assessed with a 6‐min walking test at inclusion and after 3 and 12 months within the programme.

To evaluate the feasibility of the model programme, a qualitative group interview was conducted at the end of the programme. In addition, qualitative individual interviews were conducted with all participants 3 months after they completed the programme. The interviews were conducted by the first author (LN). One interview took place in a quiet room at the primary care centre, and the other was conducted in the participants’ homes. The participants were asked to reflect on their experiences of participating in the programme, what they believed had worked well or not so well and what the programme and participation had meant to them in their daily lives. The interviews were digitally recorded and transcribed verbatim.

### 2.4 Data analysis

Due to the small number of participants, descriptive measures (medians) were used to evaluate the possible effects of the programme. At the end of the intervention, the participants were invited to attend a focus group interview. The interview lasted for 1 hr, and the content was summarized (due to small number of participants). In addition, individual interviews were conducted 3 months after completion of the intervention with the participants who had completed the model programme. The interviews lasted between 27–47 min. The manifest content was analysed in accordance with Graneheim and Lundman (2004). Thus, meaning units were identified. Then the meaning units were condensed and coded. The codes were sorted into categories.

### 2.5 Ethics

Research Ethics Committee approval was obtained from the Regional Ethics Board.

## 3 RESULTS

Four men and three women aged 51–89 years (median 80) were included. One participant withdrew after the first group session. The reason for withdrawal was that the patient anticipated that participation would be distressing. Another participant abstained from the group sessions for personal reasons but completed the programme otherwise. Three participants already had a relatively active lifestyle, while three participants had a more sedentary lifestyle. Two participants were working at baseline, while the others were retired. All were native Swedes.

The median value for the participants’ self‐efficacy in physical activity changed from 25 at baseline to 30.5 3 months after the intervention (see Table 2).

**Table 2:** Main results (medians) for the self‐report questionnaires. The scale for each measure within parentheses

	Baseline (*N* = 7)	3 months (*N* = 6)	6 months (*N* = 5)	12 months (*N* = 6)	15 months (*N* = 6)
Self‐efficacy, total score (low self‐efficacy 10‐40 high self‐efficacy)	25	23	28	28.5	30.5
Level of activity (low activity level 1‐7 high activity level)	3	5	3	4.5	4
Quality of life (worst quality of life 1‐10 best quality of life)	8	8	8	8	7.8
Fatigue (unaffected 1‐4 in rest)	2	2	2	2	2
Dyspnoea (unaffected 1‐4 in rest)	2	2	2	2	2
Health‐related quality of life, total score (no problems 5‐15 extreme problems)	6^a^	7	5	6.5	6
Little interest or pleasure doing things (not at all 1‐4 nearly every day)	2	1.5	1	1	1
Feeling down, depressed or hopeless (not at all 1‐4 nearly every day)	1	1.5	1	1	1
6‐min walk test (metres; median)	376	467	—	498.5	—

^a^ Due to technical problems, one item was missing at baseline.

The median for the participants’ level of activity increased from 3 (“I have performed light activities for approximately 30 min a day during the last week”) at baseline to 5 (“I have performed activities that have made me warm/slightly sweaty at least twice during the last week”) after 3 months within the programme. In turn, 3 months after the intervention, the participants’ median value was 4 (“I have performed activities that have made me warm/slightly sweaty at least once during the last week”; Table 2).

Concerning health‐related quality of life, a difference was found for the question about mobility. The median decreased from 2 (“I have slight problems walking about”) at baseline to 1 (“I have no problems walking about”) 3 months after the intervention.

The median number of days during the last 2 weeks that the participants had perceived little interest or pleasure in doing things decreased from 2 (“several days”) at baseline to 1 (“never”) 3 months after the intervention (Table 2).

The median walking distance at the 6‐min walk test changed from 376 m at baseline to 498 m at the end of the intervention. The median for leg muscle fatigue after the 6‐min walk test decreased from 3 on the Borg scale at baseline to 1.75 at the time of programme completion.

### 3.1 Qualitative findings

Only two participants attended the focus group interview. A summary of the manifest content showed that they perceived that the model programme had been very supportive. One participant wished it would have continued, but the other participant perceived the length of the programme to be ideal. Both participants agreed they had been motivated to perform physical activity, that they had learned a large amount of information and that meeting fellow patients had been very supportive. It had been disadvantageous that some dates had been changed and the wrist‐worn activity tracker was only perceived as initially helpful.

The analysis of the individual interviews with the six participants that completed the programme identified three categories, that is the participants perceived that the model programme implied *learning*, *motivation* and *support*. Regarding separate components (prescribed individualized activities; wrist‐worn activity tracker; activity diary; group sessions; lectures/themes at group sessions; *Active with heart failure* and individual appointments with health professionals), the group sessions and the prescribed individualized activities were most appreciated. The least appreciated component appeared to be the activity diary.

## 4 DISCUSSION

This evidence‐based one‐year model programme turned out to be feasible and relatively simple to accomplish. The participants’ self‐efficacy in physical activity and other measured variables seemed to improve, and the programme to imply learning, motivation and support was perceived by both participants who already had an active lifestyle and participants with a more sedentary lifestyle.

Most included components were appreciated by all participants. The least appreciated component was the activity diary. This was probably because it was not followed up by any health professional. The intent was that the participants should be motivated to sustain or increase their daily activity by noting number of steps. However, this metric was not followed up. As humans, we appreciate and are motivated by positive response and as no response was provided, the motivation to complete this task probably failed. The preceding scoping review identified motivating factors, such as patient education and different kinds of support or responses by email, Internet forums, Facebook groups, Instagram accounts, Snapchats or text messages (Deka et al., 2016). Such responses can also include physical activity charts or goal‐setting worksheets (Tierney et al., 2012). However, due to the existing resources at the primary care centre such components were not included.

Previous reviews (Deka et al., 2016; Tierney et al., 2012) have shown that interventions that are underpinned by a recognized theoretical framework are more likely to be effective. In part, the current study was based on theoretical assumptions about self‐efficacy. However, one weakness was that the members of the interprofessional team did not receive any guidance about the theory or how to implement it. In a future RCT, this needs to be acknowledged and healthcare professionals involved in similar projects should be educated and guided to better support participants. In turn, it is possible that if the healthcare professionals had been more engaged in the theory of self‐efficacy, the current participants’ self‐efficacy would have improved even more.

During the project, some practical problems occurred. The physiotherapist was replaced twice, and some of the invited experts cancelled on short notice. Furthermore, the individual appointments with health professionals did not work optimally because of the heavy workload at the primary care centre. The participants did not prefer this outcome, but they claimed it had not affected their motivation to sustain their level of physical activity and they perceived they learned from the programme and that it had been supportive.

The time allocated for each group session varied between 1–2 hr. Some of the participants believed that a length of 2 hr was excessive. On the other hand, some of the participants thought that this length of time was ideal. Similarly, some of the participants perceived that one year was too long, while some of the participants had wished for the project to continue for more than 1 year.

### 4.1 Strengths and weaknesses

One strength of this project was that the main researcher (LN) had an established relationship with the health professionals involved. This relationship ensured open communication based on mutual trust between the researcher and the interprofessional team. Another strength was that method triangulation was used for evaluation. To have both measurable factors and access to the participants, reflections in the interviews are considered to support the conclusions about the feasibility of this model programme.

One obvious weakness was the small sample size even for being a feasibility study, which makes it impossible to generalize the results. On the other hand, the sample size was determined in agreement with the health professionals at the primary care centre who, at the time, were under great pressure because of a heavy workload. Under these circumstances, the sample size was considered sufficient for a feasibility study. However, based on the results from this feasibility study and similar previous studies (e.g. Pozehl et al., 2010), heart failure patients’ exercise self‐efficacy can be expected to improve with 25%–30% when participating in an exercise intervention like this. Thus, to reach adequate study power (alpha 0.05, beta 0.1, power 0.9) a future RCT allowing for a 10% drop‐out rate should include at least 68 participants (34 in each group).

### 4.2 Clinical implications

This evidence‐based structured one‐year model programme can be rather easily implemented in primary care centres. However, the programme demands that health professionals have the time to fulfil their assignments. In addition, all components within the programme should be followed up. The group sessions and the lecturers and the prescribed individualized activities were highly appreciated and should be included. The activity diary and the wrist‐worn activity tracker can possibly serve as motivating factors; however, this needs to be further investigated.

## 5 CONCLUSIONS

The results of this study indicate the feasibility of this evidence‐based structured 1‐year model programme that aimed to sustain/increase motivation to engage in physical activity among heart failure patients. The patients perceived that the programme implied motivation, learning and support. It is important that all components of a programme are followed up and it is equally important that involved health professionals are allocated sufficient time, education and support to act as motivating coaches for the patients. Although no firm conclusions can be drawn about the programme's impact on participant health in the long run, the results can serve as a foundation for a future RCT. In a future RCT, activity diaries and activity trackers should be followed up.

## CONFLICT OF INTEREST

The authors declare no conflicts of interest. All listed authors meet the authorship criteria, and all authors agree with the content of the manuscript.
